# Text mining analysis to understand the impact of online news on public health response: case of syphilis epidemic in Brazil

**DOI:** 10.3389/fpubh.2023.1248121

**Published:** 2023-11-01

**Authors:** Rafael Pinto, Juciano Lacerda, Lyrene Silva, Ana Claudia Araújo, Raphael Fontes, Thaisa Santos Lima, Angélica E. Miranda, Lucía Sanjuán, Hugo Gonçalo Oliveira, Rifat Atun, Ricardo Valentim

**Affiliations:** ^1^Department of Informatics and Applied Mathematics, Federal University of Rio Grande do Norte, Natal, Brazil; ^2^Laboratory of Technological Innovation in Health (LAIS), Federal University of Rio Grande do Norte, Natal, Brazil; ^3^Information Systems Coordination, Federal Institute of Rio Grande do Norte, Natal, Brazil; ^4^Department of Social Communication, Federal University of Rio Grande do Norte, Natal, Brazil; ^5^Federal Senate, Brasília, Brazil; ^6^Ministry of Health, Brasília, Brazil; ^7^Postgraduate Program in Infectious Diseases, Federal University of Espírito Santo, Vitória, Brazil; ^8^Department of Social and Cultural Anthropology, Autonomous University of Barcelona, Barcelona, Spain; ^9^Centre for Informatics and Systems of the University of Coimbra (CISUC), Department of Informatics Engineering (DEI), University of Coimbra, Coimbra, Portugal; ^10^Health Systems Innovation Lab, Harvard T.H. Chan School of Public Health, Harvard University, Boston, MA, United States; ^11^Department of Global Health and Population, Harvard T.H. Chan School of Public Health, Harvard University, Boston, MA, United States; ^12^Department of Biomedical Engineering, Federal University of Rio Grande do Norte, Natal, Brazil

**Keywords:** communication, mass media, data mining, text extraction, public health, notifiable disease, syphilis

## Abstract

**Background:**

To effectively combat the rising incidence of syphilis, the Brazilian Ministry of Health (MoH) created a National Rapid Response to Syphilis with actions aimed at bolstering epidemiological surveillance of acquired, congenital syphilis, and syphilis during pregnancy complemented with communication activities to raise population awareness and to increase uptake of testing that targeted mass media outlets from November 2018 to March 2019 throughout Brazil, and mainly areas with high rates of syphilis. This study analyzes the volume and quality of online news content on syphilis in Brazil between 2015 and 2019 and examines its effect on testing.

**Methods:**

The collection and processing of online news were automated by means of a proprietary digital health ecosystem established for the study. We applied text data mining techniques to online news to extract patterns from categories of text. The presence and combination of such categories in collected texts determined the quality of news that were analyzed to classify them as high-, medium-and low-quality news. We examined the correlation between the quality of news and the volume of syphilis testing using Spearman’s Rank Correlation Coefficient.

**Results:**

1,049 web pages were collected using a Google Search API, of which 630 were categorized as earned media. We observed a steady increase in the number of news on syphilis in 2015 (*n* = 18), 2016 (*n* = 26), and 2017 (*n* = 42), with a substantial rise in the number of news in 2018 (*n* = 107) and 2019 (*n* = 437), although the relative proportion of high-quality news remained consistently high (77.6 and 70.5% respectively) and in line with similar years. We found a correlation between news quality and syphilis testing performed in primary health care with an increase of 82.32, 78.13, and 73.20%, respectively, in the three types of treponemal tests used to confirm an infection.

**Conclusion:**

Effective communication strategies that lead to dissemination of high quality of information are important to increase uptake of public health policy actions.

## 1. Introduction

**Figure 1:**
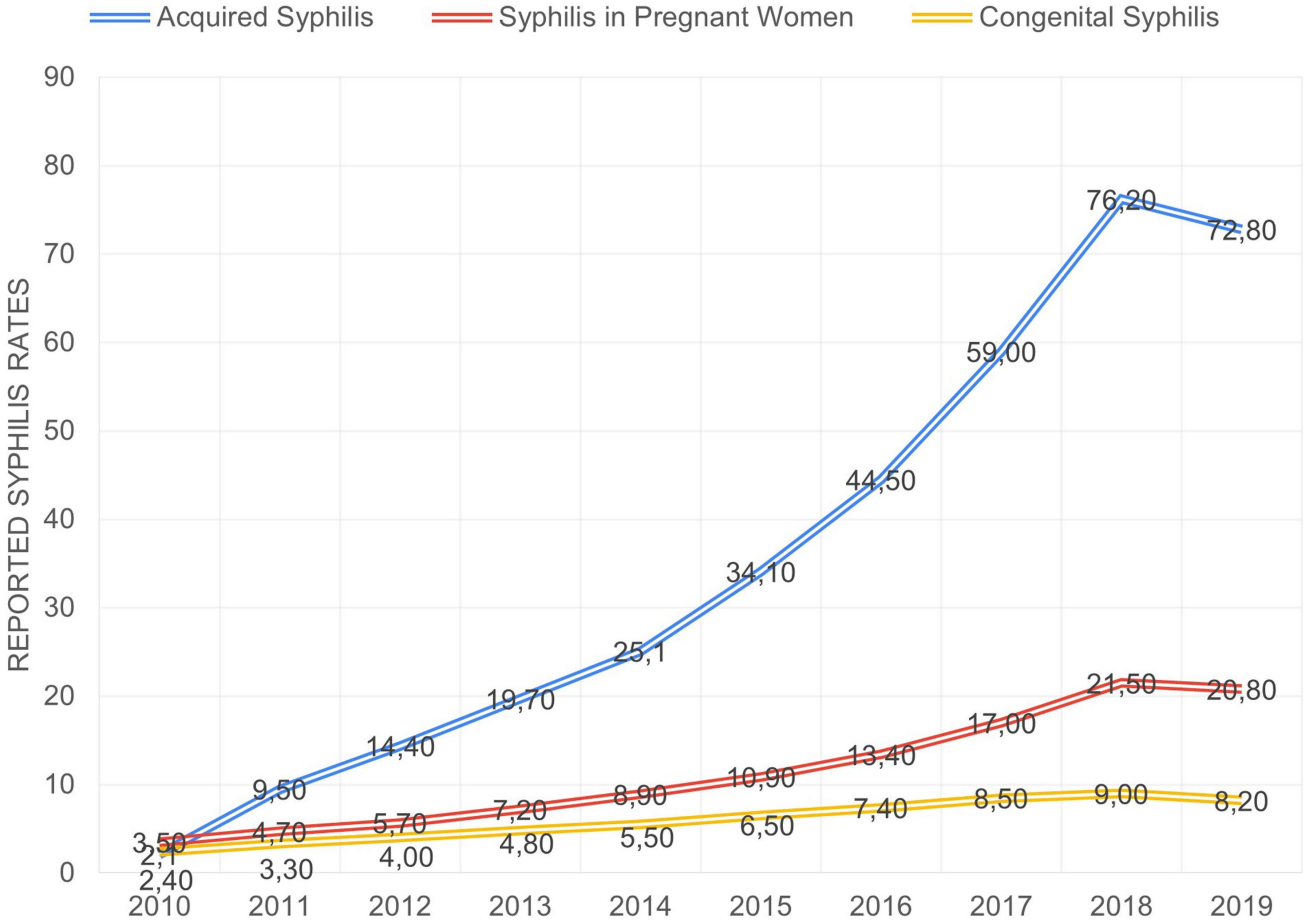
The incidence of syphilis in Brazil from 2010 to 2019.

Syphilis is a major public health problem in Brazil, an upper-middle income country with a unified health system and universal health coverage (1). Figure 1 shows the evolution of syphilis rates from 2010 to 2019. During this period, the incidence rate of congenital syphilis reached, in 2018, 9.0 cases per 1,000 live births, decreasing to 8.2 cases per 1,000 live births in 2019. The detection rate of syphilis in pregnant women reached 21.5 cases per 1,000 live births in 2018, and in 2019 it decreased to 20.8 per 1,000 live births. Acquired syphilis, listed as a notifiable disease in 2010, reached 76.2 cases per 100,000 population in 2018, but reduced to 72.8 cases per 100,000 population in 2019 (2).

In 2016, the Brazilian Ministry of Health (MoH) declared syphilis, a sexually transmitted infection, a public health emergency in Brazil and created a national initiative, “Applied Research for Intelligent Integration to Strengthen Healthcare Networks for a Rapid Response to Syphilis” — also known as the “Syphilis No!” Project (SNP) – to combat syphilis. The Laboratory for Technological Innovation in Health (LAIS) partnered with the MoH and Federal University of Rio Grande do Norte (UFRN) to implement the Syphilis No! Project.

The Syphilis No! Project comprised academic and medical research, as well as educational and health communication activities consisting of corporate and digital communication, advertising, and public relations. Panel 1 describes the core components of the Syphilis No! Project.

**Table:** 

**Panel 1 – Syphilis No! Project** The Syphilis No! Project is a national rapid response strategy to syphilis of the National Health System of Brazil (SUS). The Project is primary health care-oriented and aims to promote collaborative actions between surveillance and care in the territory under an interfederative perspective of technical cooperation in health. Syphilis No! operationalization: Line 1 – Actions of universal scope: Purchase and distribution of crystalline and benzathine penicillin; Purchase and distribution of rapid syphilis tests; Strengthening of laboratory structure for diagnosis; Instrumentation of situation rooms in all Brazilian districts and in the Federal District; Implementation of national prevention campaigns; Development of education and communication tools to be made available to all municipalities; Dissemination of strategic information to municipal and district managers to support decision-making; Development of studies and research aimed at coping with syphilis. Line 2 – Actions developed mainly by Research and Intervention Supporters on priority areas selected by epidemiological criteria (capitals and strategic municipalities, a total of 100, that in 2015 represented 68.95% of congenital syphilis burden of disease in Brazil) (3). Interfederative technical cooperation for implementation of committees (municipal/regional) to investigate mother-to-child transmission of syphilis; Cooperation to evaluate actions to confront Syphilis in the municipal and district plans, health programs and management reports of the priority districts and municipalities; Interfederative technical cooperation for implementation of syphilis epidemiological surveillance rooms at the municipal level; Interfederative technical cooperation to strengthen the health care network and different spaces of care provision for implementation of syphilis care lines (syphilis in pregnant women and sexual partners, children exposed to maternal syphilis, and acquired syphilis), also with intervention in key populations (sex workers, gay persons and men who have sex with men, transgender people); Interfederative technical cooperation to strengthen intersectoral actions in the territory, prioritizing social control (induction of agendas involving health, education and social assistance); Interfederative technical cooperation to monitor the development of project actions in the situation rooms.

The Syphilis No! Project also developed a digital health ecosystem, namely Hermes, with analytics underpinned by computational tools and machine learning that integrated data from multiple sources to monitor project implementation and campaign progress by measuring education and communication activities and through epidemiological surveillance on the number of tests and number of syphilis cases (Panel 2 in Methods section).

In 2018, the 2018–2019 National Campaign to Combat Syphilis (the Syphilis No! Campaign) was launched with the theme “test, treat, and cure” and the motto “remember to take care.” Aired between November and December 2018, the campaign was the first to involve national mass media outlets as part of an integrated response with local actions in priority areas, that had high rates of syphilis (3–5). In January to March 2019, the SNP implemented syphilis-related actions through digital social networks. A key theme of the communication campaign developed by the “Syphilis No” project was “Test, Treat and Cure.”

During the “Syphilis No!” Campaign, the media played a vital role in enhancing understanding of the disease through messages in several channels. The organizers produced and disseminated a large amount of material throughout Brazil. The campaign broadcasted videos about syphilis via television; radio and streaming platforms delivered pre-recorded audio messages (spots and testimonials); sponsored videos on Youtube channels and pre-recorded audio messages were delivered as ads on Spotify; ads were printed in newspapers and consumer magazines; posters were displayed in shop windows, bus stops, billboards, and other forms of media were implanted in publicly accessible urban spaces; Messages were posted on social networking sites (Facebook, Instagram, and Twitter) by influencers.

The media, especially the press, play a pivotal role in selecting and creating messages to represent facts (6, 7), promoting public health, influencing public opinion, and generating awareness about health issues. Both in content and form, news stories paint a picture of the ‘real world,’ whose framing, through narrative conventions, can be refined through topics of discussion to social conversations (8). The press and news media report on matters of interest to the public sphere not necessarily to tell people ‘how to think,’ but ‘what to think about’ (9).

This paper relies on the premise that a nationwide public communication campaign will likely rely on news coverage (10). Campaigns aim to exert direct influence on those identified as target population by delivering evidence-based health information and addressing disinformation and misinformation. However, experience with political campaigning has shown that capturing the media’s attention is the immediate outcome, and “[i]mplicit in this campaign perspective is the idea of the public agenda because control of media setting implies significant influence over the public’s agenda” (9).

The prominence of a topic in the media’s agenda creates ‘salience’ (9, 11, 12). In other words, a topic is highlighted in a given period compared to others based on one or more attributes, according to the framing by the media (9, 11, 12) through a gatekeeping process, which selects the facts that become news and are disseminated by media outlets (9, 13).

In addition, in a systematic literature review performed by our research group, we observed a gap in assessing the impact of public health campaigns, regarding the use of online data (i.e., online news) and others user-generated Internet content (14). Thus, this study analyzes online news on syphilis in Brazil by mapping key elements of the news posted online between 2015 and 2019 and measuring their quality. The study covers two time periods: (1) 2015 to 2017, related to communication actions prior to the Syphilis No! Project, and (2) 2018 to 2019, related to the news coverage of communication actions carried out during the Syphilis No! Project.

In this study, we hypothesized that the quantity and quality of news about syphilis disseminated in the form of earned media are significant indicators for measuring the impact of communication actions developed by the Syphilis No! Project throughout 2018 and 2019. Earned media can influence the public agenda and people’s decision-making (9, 11, 12) due to the breadth of timely and sound information provided to the general public. Indicators related to earned media coverage could be used to measure the success of a public health campaign (15, 16), to examine the effect of communication actions (17, 18) and to improve public policy and public health interventions.

## 2. Materials and methods

We conducted a field investigation before characterizing and qualifying news through Hermes. The idea was to outline quality indicators of information about syphilis in the media agenda. This step identifies a type of attribute (9, 11, 12) about the framing of reported news. We determined the indicators, then extracted and performed a content analysis of the web pages selected. The workflow performed in this section is presented in Appendix Figure A1.

We used the Google search when identifying the relevant web pages, using the keywords: ‘syphilis,’ ‘syphilis campaign,’ ‘AIDS,’ ‘AIDS campaign,’ and ‘sexually transmitted diseases.’ We narrowed the search to documents written in Brazilian Portuguese. From the search results, two researchers, specialists in public health communication, manually accessed the most relevant web pages and selected 153 texts, that were characterized as news (19), based on elements that constitute a news piece, namely, the headline, the lead (the paragraph that summarizes the “who, when, where, what, why, and how”), and the body, which further elaborates on the elements mentioned in the lead.

In order to map out relevant features in the news identified, we used thematic analysis and axial coding to identify emerging themes and sub themes (20). Through indexicality, that is, the process of analyzing text fragments that constituted the news that were analyzed, we categorized, by induction, news about syphilis into the following exploratory categories (or quality indicators): definitions of syphilis; epidemiological data on syphilis; how to prevent syphilis; how or where to get a syphilis rapid test/diagnosis; consequences of syphilis in key populations (such as pregnant and infants) and risk of lethality due to tertiary syphilis; effective treatments to cure syphilis; and public communication campaigns (details provided in the Appendix Topic A2).

This analysis resulted in seven generic news categories: Disease Definition; Epidemiological Indicators; Prevention; Rapid Test/Diagnosis; Consequences; Campaign, and Treatment. Table 1 provides an overview of the exploratory categories and their quantitative distribution of text fragments across the body of the 153 news analyzed. These text fragments will be defined as ‘training data’ and after homogenization and standardization, they will serve to verify if the most important words of each category are contained in the online news.

**Table 1:** Quantitative distribution of exploratory categories found in the training data from the search results performed by researchers.

Exploratory categories	Generic term	Number of text fragments in the news
Definitions of syphilis	Disease definition	90
Epidemiological data on syphilis	Epidemiological indicators	96
How to prevent syphilis	Prevention	73
How or where to get a syphilis rapid test/diagnosis	Rapid test/diagnosis	76
Consequences of syphilis in key populations and the risk of lethality from tertiary syphilis	Consequences	75
Public communication campaigns	Campaign	65
Effective treatment for syphilis	Treatment	68

In addition, we designed an online survey on the identified exploratory categories and recruited seven specialists in communication and public health to analyze the importance of their presence in news items as a way of qualifying texts about health problems. The evaluators had experience in the following areas: federal health management; state health management; primary care professional or municipal management; management in the Health Council; university research; communication and health; and journalism. Each question individually evaluated the importance of the categories through a balanced Likert Scale as follows: not important; slightly important; moderately important; very important; and extremely important.

**Figure 2:**
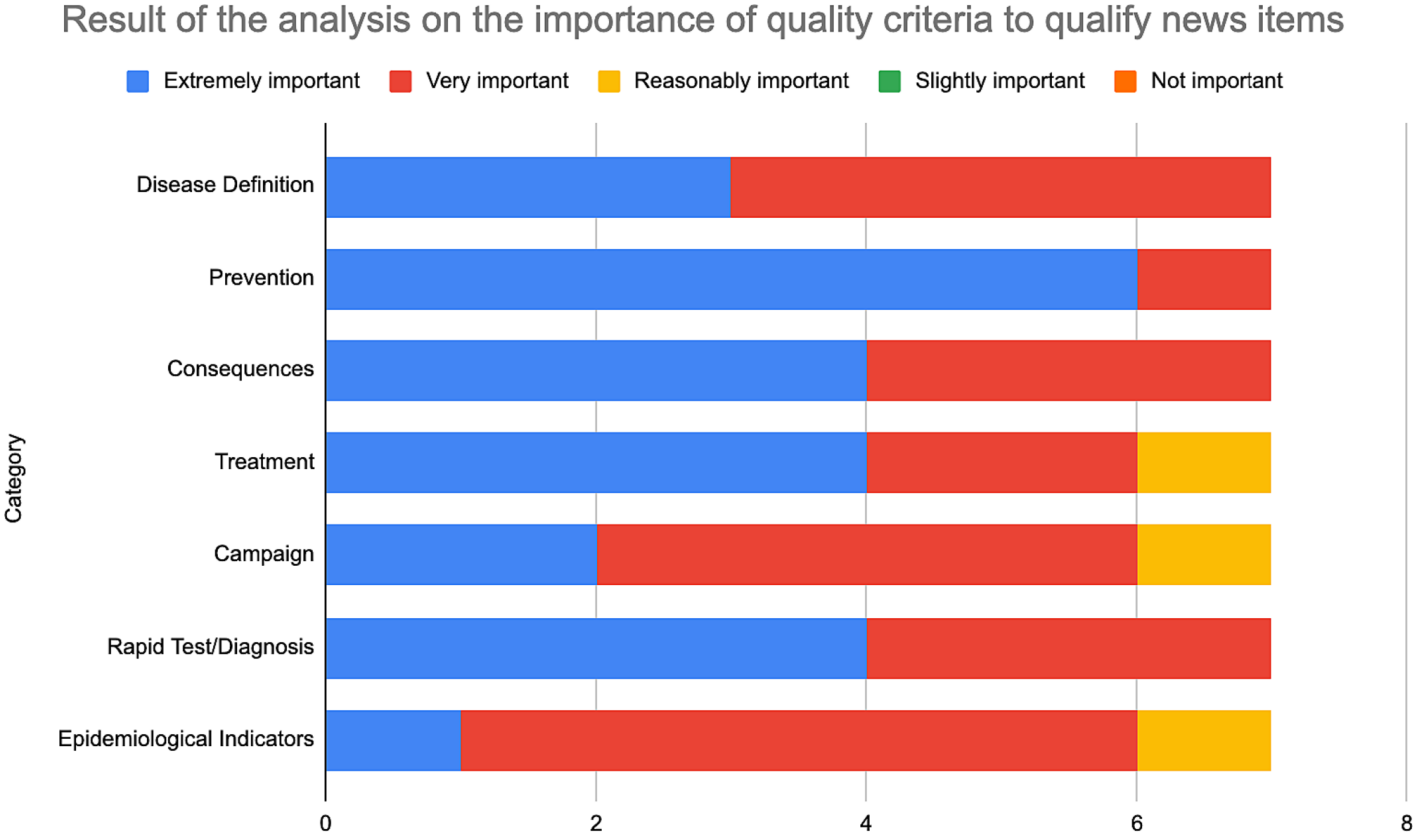
Findings of the analysis on the significance of quality criteria for evaluating texts related to health issue.

Figure 2 highlights the varying levels of importance assigned to different categories related to the news items. Categories such as prevention, treatment, and consequences are consistently rated as highly important, while others, such as disease definition and rapid test/diagnosis, also hold significant relevance. This assessment supports our findings regarding the importance of qualifying health news using the defined criteria.

Once the exploratory categories and training data were defined, we proceeded to the second step of the workflow, which consisted of identifying the volume and quality of online news about syphilis through Hermes.

Based on a multidimensional analysis framework, such an ecosystem monitors the reach of public health actions related to campaign activities, education, communication, and epidemiological surveillance (4). It used computational tools and machine learning to monitor progress with Syphilis No! project and assist managers and decision-makers in evaluating the effects of public health policy interventions and communication campaigns. Panel 2 describes the elements, the computational tools and machine learning approaches used for analysis in the Hermes ecosystem with more details provided in the Appendix.

**Table:** 

**Panel 2: Hermes – A Digital Ecosystem to Assess Public Health Policies** A software ecosystem refers to a collection of software products that have some given degree of symbiotic relationships (21), used by a set of actors on top of a common technological platform (22). A software ecosystem must be seen from multiple perspectives, such as technology, communication, and epidemiological surveillance. Hermes focuses on bringing knowledge to stakeholders through the heterogeneous data collection, processing, integration, and visualization of this information through a single platform, using computational tools, machine learning techniques, and statistical analysis that enable public health policy evaluation. **Features:** **Actor-Centered** It recognizes the different actors, e.g., stakeholders, decision-makers, communication campaign analysts, and financial and public managers. **Data-Driven** Enables collecting heterogeneous data and processing it through computational techniques using machine learning. Facilitates data interpretation, transforming it into feasible information for assessment. Strengthens decision-making based on prediction and understanding of a problem. **Heterogeneous Data Sources and Data integration** Hermes collects and integrates several data sources, such as Google Search, Google Trends, epidemiological surveillance databases of the Ministry of Health, databases of Massive Open Online Courses (MOOCs) collected from SUS’ Virtual Learning Environment (AVASUS); social networks (Instagram, Twitter, Facebook, and YouTube); in addition to allowing manual entry or upload of files comprising data from Public Health Campaigns. It ensures consistency, quality, and completeness for correlation and causality checking. **Automated Processes** It automates processes based on machine learning algorithms, natural language processing, and statistical analysis: (i) text mining and feature extraction; (ii) sentiment analysis; (iii) clustering; (iv) time series decomposition; (v) interrupted time-series segmented regression analysis; and (vi) coefficient correlation analysis between variables of interest.

In this study, from January 2015 to December 2019, Hermes collected online content that was Google indexed and included the term ‘syphilis’ in Brazilian Portuguese web pages. It performed this task by using a Google Search API that allows retrieving, in JSON format, a set of websites indexed by keyword searched for (23). The fields returned by the automatized search results included: the date when the news was indexed, URL, title, and a snippet of the news. 1,049 web pages were found in this period using these parameters.

Hermes has a content extraction module that uses the Newspaper Python library (24) and extracted the following fields from each collected URL: title, publication date, keywords, running text, and abstract.

At this point, it was necessary to identify if these 1,049 web pages were, in fact, news. Thus, we defined a typology that made it possible to identify which web pages featured online news. A manual identification was carried out and 630 web pages were defined as news (Table 2).

**Table 2:** Web pages categorized after main text content extraction.

Type	Rationale	Number
Page not found / Content not available	Page no longer exists, or content is exclusive to members.	42
Search results	Page content results from a search within the website.	69
Non-text content	Page does not include text content (e.g., podcasts, images, videos, PDF, or PPT files).	123
Scientific research	The main content is part of a scientific research summary, such as an article, poster, thesis, or dissertation.	185
News	The main content has characteristic features of a news report.	630

To substantiate and build the automated extraction of features found in the online news through Hermes, we used the text mining technique, otherwise known as Text Data Mining, which calculates the weight of a keyword (score) in a vector model (matrix) (25).

We checked unigrams (individual terms), bigrams (two consecutive terms), and trigrams (sequences of three terms) through a comparative analysis to ascertain which keywords combination could be the most suitable for extracting characteristic features included in news. We chose to use bigrams since they provide a richer contextual representation than unigrams, as they capture the proximity relationship between adjacent words and have a smaller dimensionality than the trigrams. It allowed a more refined analysis of news texts’ specific characteristics and contexts. For example, a bigram like “syphilis campaign” can convey more accurate and meaningful information than the isolated terms “syphilis” or “campaign.” The choice of bigrams is directly aligned with the study’s objectives, which aimed to identify specific characteristics (exploratory categories) of news items. By capturing meaningful relationships between adjacent words, bigrams allowed for extracting more accurate and relevant characteristics, providing valuable insights into news content.

The following procedures were executed in order to homogenize and standardize the bigrams: (i) changing the capitalization of words to lowercase, (ii) deleting special characters, (iii) removing all punctuation, (iv) removing extra spaces, (v) removing accents and numbers, and (vi) deleting “stop words” (words that had no meaning, such as adverbs and prepositions). Finally, the sentences were converted into bigrams, which comprise a dictionary of words representing each group (the training sample and the online news about syphilis). Table 3 provides examples of the main bigrams found for each exploratory category in the training sample.

**Table 3:** Main bigrams found for each exploratory category in the training sample.

Category	Bigrams
Disease definition	Bacteria cure; sexually transmitted; transmitted caused; curable treatment; simple treatment; manifest infection; sexually disease; called syphilis
Epidemiological indicators	Year age; health increase; new cases; cases type; congenital period; types syphilis; syphilis parents; thousand people; last bulletin
Prevention	Condom distribution; better condom; through use; prevention use; doctors reinforce; need condoms; reinforce need; use condoms
Rapid test / Diagnosis	Serologic test; rapid test; syphilis result; test detected; easily detected; rapid used; used diagnosis; result minutes
Consequences	Case not; baby development; cause complications; lead death; genital sores; genital rashes; cause abortion
Campaign	Syphilis campaign; no syphilis; municipal campaign; activities integrate; campaign ending; campaign intended
Treatment	With penicillin; penicillin treatment; based antibiotics; base treatment; antibiotic treatment; treated syphilis

This process yielded a matrix, with each row corresponding to a news item and each column to the bigrams. The cells refer to the score of bigram’s relevance in the news, and they were estimated through Term Frequency – Inverse Document Frequency (TF–IDF) (26). This technique statistically determines the importance of a word in a document corpus relative to other texts within the same database. The weight of a word for such a document is contingent on the number of times it appears in it, but is offset by the frequencies of the words in the other documents within the same database (27). So, words that are common in every document, such as *this*, *what*, and *if*, rank low even though they may appear many times, since they do not mean much to that document in particular.

The TF–IDF used to fit these data is straightforward.

$TF-IDF\left(t,d,D\right)=TF{\left(t,d\right)}^{*}\;IDF\left(t,d,D\right)$

Where the Term-Frequency (TF) measures up to how many times (freq) the word t exists in the document d. This frequency is calculated as follows:

$TF\left(t,d\right)=\mathrm{freq}\left(t,d\right)$

For example, in this collection of documents: “this is the first document.,” “this document is the second document.” and “Is this the first document?,” the TF of the word document is 1 for the first document, 2 for the second document and 1 again for the third document.

The IDF is used to measure the t score from the frequency in d and in the collection of documents. The IDF is defined by the log between the total of documents (N) and the frequency of documents d where the term t occurs (dft). This frequency is calculated as follows:

$$IDF\left(t,d,D\right)=\log\left(\frac{N}{dft}\right)$$

Using the same example, the IDF of the *t document* is log(3/3), log(1), thus the IDF of the *t document* is 0. Therefore, the final score of *t* is the weight resulted by frequency the *t* in the *d* (TF) and the inverse document frequency (IDF).

Subsequently, all scores of bigrams found within news’ characteristic features were summed, then a final score was defined. Finally, considering the results, the Hermes ecosystem, in an automated process, determined the acceptance threshold for category identification for each of the seven categories identified, based on the category’s average score.

The acceptance threshold is responsible for guaranteeing that one news holds the minimum score for the presence of features to be identified. Thus, we verified whether or not the score applied is greater than or equal to the acceptance threshold. If so, the category identified characterized such a news. This procedure was used for all 630 news extracted and for all seven categories we defined, in which a news can include none, one, or more exploratory categories.

Quality parameters of news were determined based on the presence and combination of categories in them, as follows: low quality (0–2 categories), medium quality (3–4), high quality (5 or more).

Using Spearman’s Rank Correlation Coefficient (28), we examined the association between the number of news over the period January 2015 to December 2019, grouped by their respective quality levels (low, medium, and high), and the number of serology tests for syphilis diagnosis performed in primary health care in Brazil. The data on number of tests were obtained through the Outpatient Information System (Sistema de Informação Ambulatorial, SIA) of the Unified Health System (Sistema Único de Saúde, SUS), available on the MoH webpage.^1^

SIA/SUS allows for the retrieval of test results based on monthly and yearly quantities, as follows: (i) Treponemal test for syphilis detection, (ii) Fluorescent Treponemal Antibody-Absorption (FTA-ABS) IgG test for syphilis diagnosis, (iii) Fluorescent Treponemal Antibody-Absorption (FTA-ABS) IgM test for syphilis diagnosis, (iv) Nontreponemal test for syphilis detection, (v) Nontreponemal test for detecting syphilis in pregnant women, (vi) Rapid Syphilis Test, (vii) Rapid syphilis test for detecting the infection in pregnant women or fathers/partners. The collected data for testing includes the period from 2015 to 2019.

We also repeated the analysis using Pearson’s and Kendall’s coefficients, but Spearman’s Rank Correlation Coefficient method (also referred to as Spearman’s rho) revealed better results. Perhaps because (i) data are generally not distributed across the two variables, (ii) there is a monotonic relationship among data, and (iii) both variables are ordinal (29).

## 3. Results

A total of 1,049 web pages were gathered through the utilization of the Google Search API. Among these, 630 pages were classified as earned media. An upward trend in the quantity of syphilis-related news item was observed over the years, with 18 articles in 2015, 26 in 2016, and 42 in 2017. However, a significant surge in news coverage occurred in 2018 (107 articles) and 2019 (437 articles). Despite this increase, the proportion of high-quality news remained consistently high, with 77.6 and 70.5% respectively, aligning with previous years. Our findings indicated a correlation between news quality and the performance of syphilis testing in primary healthcare settings, demonstrating an increase of 82.32, 78.13, and 73.20%, respectively, across the three types of treponemal tests used to confirm an infection.

Table 4 provides the total number of news mapped by year, along with exploratory categories found. The number of news increased gradually from 18 in 2015 to 26 in 2016 and 42 in 2017, then rose substantially to 107 in 2018. In 2019 the number of news increased to 437 – a figure higher than the sum of the number of news in the four preceding years.

**Table 4:** Distribution of exploratory categories found in analyzed news on syphilis per year, from 2015 to 2019.

Exploratory category	2015	2016	2017	2018	2019
Number of online news item about syphilis per year	18 (2.86%)	26 (4.15%)	42 (6.67%)	107 (16.98%)	437 (69.37%)
Campaign	11	16	32	78	308
Consequences	15	21	41	91	352
Disease definition	11	14	26	67	241
Epidemiological indicators	13	11	29	55	243
Prevention	15	21	35	86	360
Rapid test/Diagnosis	13	25	39	92	382
Treatment	16	22	34	88	348
None of the categories	1	1	0	11	22
Total number of news	630				

**Figure 3:**
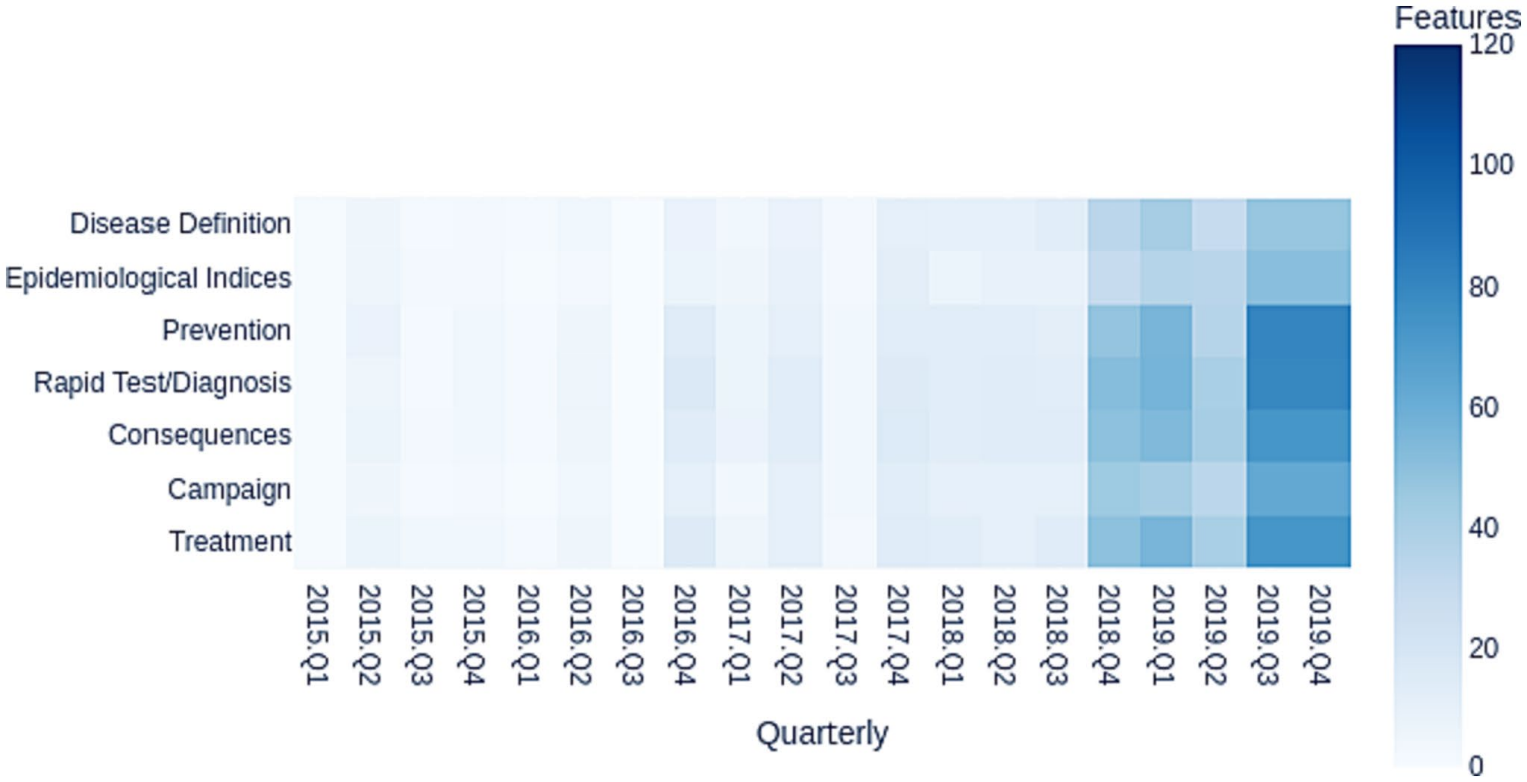
Heat map of categories by quarter from 2015 to 2019.

Figure 3 shows the mapping of categories by quarter from 2015 to 2019. Each cell represents the number of news with respective categories. In 2015 the highest number of news was in Q2 (April–June), citing the terms “prevention,” “consequences,” and “treatment.” The periods of highest intensity in 2015 is related to the festive seasons in Brazil (June festivals) and the month of October, when the Ministry of Health dedicates to discussing Sexually Transmitted Infections (STIs) and disseminates data from the epidemiological bulletin in Brazil. In 2016 and 2017 the highest number of news was in Q4 (October–December), citing the terms “campaign,” “consequences,” “test/diagnosis,” and “treatment,” coinciding with the National Day to Combat Syphilis and Congenital Syphilis in October. In the rest of the year these categories were hardly ever or never mentioned in the news. As of 2016 Q4, the patterns begin to gain greater intensity due to the Ministry of Health’s declaration regarding the syphilis epidemic in Brazil. In this way, the theme gained more space in the Brazilian media agenda than in previous years.

In 2017, the news focused on the impact of the declaration of the syphilis epidemic in Brazil made at the end of 2016. However, the campaigns were still focused on congenital syphilis, produced with low investment, and disseminated mainly on social networks. As of 2018, SNP actions were intensifying, holding seminars with state and municipal entities in all regions of Brazil on the strategy to combat syphilis. Then the national campaign was launched, maintaining greater journalistic coverage in the period.

The “Syphilis No!” Project was part of this strategy to change course in the fight against syphilis in Brazil, involving more resources for publicity campaigns, including intelligence actions that mobilized states and municipalities. Qualified actors selected in each territory acted in this project, which made up a support network for research and project actions, as detailed in Panel 1.

In 2018 and 2019, while there was a preponderance of news in Q4 of each year, and in particular October, there were news throughout the year. The majority of news mentioned “campaign,” “consequences,” “rapid test/diagnosis,” “treatment,” and “prevention.”

Table 5 shows the number of news categorized by quality. While the volume of news increased substantially in 2018 and 2019, the relative proportion of high-quality news remained consistently high (77.6 and 70.5% respectively) and in line with similar years.

**Table 5:** Number of news each year categorized by quality.

Quality level	2015		2016		2017		2018		2019	
	*n*	% of total	*n*	% of total	*n*	% of total	*n*	% of total	*n*	% of total
High	13	72.2%	19	73.1%	33	78.6%	83	77.6%	308	70.5%
Medium	2	–	2	–	6	–	10	–	71	–
Low	3	–	5	–	3	–	14	–	58	–
Total	18	–	26	–	42	–	107	–	437	–

There was a correlation between the quality of news about syphilis and the number of serology tests performed in primary health care for diagnosing syphilis as shown in Table 6. The rapid tests for syphilis in pregnant women or fathers/partners showed a 65.33% correlation with high-quality news, while rapid tests in the general population had a 58.98% correlation. Nontreponemal tests for syphilis showed a 69.15% correlation with the high-quality news and treponemal tests (confirmatory tests for syphilis) showed 82.32, 78.13, and 73.20% over the same period.

**Table 6:** Correlation between the number of tests and the number of news according to their quality.

Types of syphilis tests	News quality		
	High	Medium	Low
Treponemal test for syphilis detection	73.20%	67.15%	62.78%
Nontreponemal test for syphilis detection	69.15%	62.04%	58.66%
FTA-ABS IgG test for syphilis diagnosis	78.13%	62.12%	64.58%
FTA-ABS IgM test for syphilis diagnosis	82.32%	68.84%	68.31%
Nontreponemal test for detecting syphilis in pregnant women	64.92%	58.76%	50.85%
Rapid syphilis test	58.98%	46.31%	42.64%
Rapid syphilis test for detecting the infection in pregnant women or fathers/partners	65.33%	56.27%	52.07%

## 4. Discussion

Implementing effective communication strategies to facilitate the widespread circulation of accurate and reliable information is crucial in order to enhance the dissemination of public health interventions. Our findings demonstrate a significant surge in news coverage through earned media as well as increased testing, indicating a positive correlation between the two. This highlights the potential of continuous and comprehensive national mass media outlets as an instrument for promoting public policies addressing health crises.

The number of news on syphilis rose rapidly following the implementation of the communication strategy as part of the Syphilis No! Project. While there was a cumulative total of 86 news on syphilis in 2015 to 2017, the number of news rose to 107 in 2018 (154.8% increase compared to 2017) and 308 in 2019 (308.4% increase compared to 2018).

In 2010, syphilis became a notifiable disease in Brazil and was declared a public health emergency in 2016. However, these major public health policies did not lead to a change in the number of news or an increase in testing and diagnosis (2, 30).

While in May 2015, the MoH conducted a campaign on mother-to-child transmission (MTCT) for Mother’s Day and in 2016 and 2017 ran communication campaigns on the National Day to fight Against Syphilis and Congenital Syphilis, these were one-off events (31) and had no meaningful effect on news and testing.

The launch of the communication actions of the Syphilis No! Project in February 2018 coinciding with the Brazilian Carnival was a turning point. A campaign on social networks with local and state initiatives, followed. In March 2018, the SNP launched intervention agendas across the country through the Ministry of Health, with an emphasis in 100 priority municipalities (3, 4, 32). In November 2018, the national campaign “Remember to take care of yourself” (2018–2019) was launched. The communication campaign meant that actions were not have meant that communication was no longer limited to one-off annual events, such as the National Day to fight Against Syphilis and Congenital Syphilis, but were spread throughout the year (31) involving a myriad of products and communication actions, including in ‘paid and owned media’ that helped to increase news in earned media.

Carrying out media campaigns in support of the adoption and dissemination of public health interventions is a necessary condition to generate in society a regime of attention and visibility for a problem such as syphilis. The media can generate a legitimation process and make the problem visible on a national scale. However, to raise awareness and change habits in the population, developing a set of articulated actions in the territories is necessary.

Notably, public policies to combat syphilis had no significant effect between 2010 and 2016, clearly observed by the increase in cases and the low amount of news on the subject in recent years (2015–2017). However, as of 2018, it is possible to observe the influence of communication efforts through the support of the theme in media coverage.

It is important to emphasize that the SNP intervention actions go beyond the communication area since actions developed mainly by Research and Intervention Supporters in priority cities with a high rate of cases of congenital syphilis helped to: strengthen the health care network and the different care spaces for the implementation of syphilis care lines; implement syphilis epidemiological surveillance rooms at the municipal level; evaluate actions to combat syphilis at municipal and district levels. The Research and Intervention Supporters whose actions helped improve coordination of communication efforts and the messaging among state and local health offices and the local press, acting as an “opinion leader,” to establish a “two-step flow of communication” (33) to ensure high-quality news. The communication actions led by SNP produced greater “resonance,” with local and national press (17).

In addition, actions of a universal scope were also carried out throughout the territory, such as: the purchase and distribution of crystalline and benzathine penicillin; the purchase and distribution of rapid syphilis tests; reinforcement of the laboratory structure for diagnosis; and the instrumentation of situation rooms in all Brazilian districts and the Federal District.

The SNP included various actions that enhanced the impact of the communication campaign and ensured that the topic remained prominent on the agenda-setting it apart from previous years.

McCombs (9) argues that the agenda-setting process depends not only on the time period of media exposure a topic has but also on the potential correspondence to the audience’s need for orientation on the topic. The results reveal a substantial increase in news in earned media and in testing, with a correlation between the two, suggesting that a sustained communication campaign could be a powerful tool for promoting public policies to tackle health crises.

The Ministry of Health recorded the highest number of syphilis tests performed per 1,000 population in 2018 (2,1 million) and 2019 (2,5 million), compared to 1,4 million in 2017. While in 2010–2018, the number of cases of acquired syphilis, syphilis during pregnancy, and congenital syphilis increased substantially, but fell in 2019 (4, 32, 34).

Digital information and communications technologies in health and the application of computational methods based on artificial intelligence can be used to develop predictive analytics to inform real-time response to effectively manage public health outbreaks and crises. The Hermes digital ecosystem developed for the project, played a critical role in monitoring campaign progress, regularly capturing data registering, and processing of information related to communication actions, health system interventions (testing) and epidemiological parameters (number of cases of acquired syphilis, syphilis in pregnant women and congenital syphilis). The use of computational methods that enabled analysis of data of heterogeneous nature to examine the public health response and its results in real time throughout the country to provide a powerful tool in planning and monitoring of the public health campaign for syphilis and can be transferred to other public health challenges.

Nowadays, for an individual to perceive a subject as relevant, such subject needs to be highlighted in the media with a particular frequency, being highlighted in their agenda. Thus, if a subject gains greater exposure in the media for some time, it becomes seen as important by the public. For example, until 2017, syphilis was not highlighted in the Brazilian media. Therefore, it was considered a neglected disease.

As of 2018, a set of systematic and strategic communication actions has been developed, supported by technological, financial and intellectual resources. The actions guided the priority target audiences, health managers, and professionals nationwide. These actions intensively positioned syphilis in the Brazilian media agenda from 2018 onwards, drawing public attention. The lack of qualified information, as it is a neglected disease, generated a feeling of uncertainty in public, who started to seek more information and seek health units and learn about the forms of diagnosis and treatment. Notably, an effective and ongoing communication campaign can promote public policies and provide efficient responses to health crises.

There are potential limitations of our study. The first one relates to the completeness of the news collection used as a dataset, an external threat mitigated by choosing one of the largest existing content indexers, namely Google Search. However, it would be a mistake to assume that Hermes can retrieve every existing online news through the Google Search API. In addition, Hermes used filters for: (i) language, which narrows the search to documents written in Brazilian Portuguese, and (ii) geolocation, which limits the search results to documents originated from Brazil. That may constitute a barrier insofar as the geolocation parameter checks the domain (URL) and the geographical location of the Web server’s IP address. Future works may add other search engines, such as Bing and Yahoo, to expand the search result for news items on the Internet. The second limitation relates to the search results related to the 42 missing pages Google indexed. If they had been incorporated into the analysis, they would likely increase the number of resulting news. However, when analyzing the date of news of missing web pages, we observed they had the same proportionality of 2015 (*n* = 1), 2016 (*n* = 3), 2017 (*n* = 1), 2018 (*n* = 15), and 2019 (*n* = 22) results.

Notwithstanding limitation, the study reveals the effective application of a digital health system that incorporates all the elements of a complex public health campaign that included a communication campaign, education, health system interventions, training, expanded access to testing and treatment.

The ability to explore online news through machine learning has aroused the interest of parallel study groups, bringing new insights for stakeholders to analyze public health campaigns from different perspectives, such as sentiment analysis techniques (35) and similarity analysis (36).

## 5. Conclusion

This study from Brazil, an upper-middle income country, led by a multidisciplinary group of researchers involving public health specialists, clinicians, experts in computational science, data scientists, educationalists, communication experts and marketing experts reveals the successful implementation of public health actions with a communication campaign that led to major increases in online news related to syphilis and the shift in the media landscape and the public health response after the Syphilis No! Project interventions.

The Hermes ecosystem was able to effectively capture the number and frequency of news stories before and after the Syphilis No! Project, effectively classify news according to informational categories and identify high-, medium-, and low-quality news, and to examine the relationship between the communication campaign and public health results to orient more effective and targeted health policies and interventions to manage the syphilis epidemic in Brazil.

The study reveals the utility of integrated digital health information systems in guiding public health policies and actions to ensure effective responses to public health challenges across all country income categories.

## Data availability statement

The raw data supporting the conclusions of this article will be made available by the authors, without undue reservation.

## Author contributions

RP, JL, AA, LSi, and RV: conceptualization. RP, RF, JL, AA, LSi, and RV: methodology. RP, RF, and HG: software, formal analysis, and data curation. RP, JL, LSi, AA, RF, TL, AM, LSa, HG, RA, and RV: validation and writing – review and editing. RP, JL, AA, RF, LSi, and RV: investigation. RP, LSi, and RV: resources. RP, JL, AA, RF, and TL: writing – original draft preparation. RP and RF: visualization. JL, LSi, RV, and RA: supervision. RV: project administration and funding acquisition. All authors contributed to the article and approved the submitted version.

## Footnotes

^1^https://datasus.saude.gov.br/acesso-a-informacao/producao-ambulatorial-sia-sus/ (accessed on 25 November 2020).
