# Supplementary material for: Text mining analysis to understand the impact of online news on public health response: case of syphilis epidemic in Brazil
Source: Front Public Health. 2023 Nov 1;11:1248121. doi: 10.3389/fpubh.2023.1248121 (PMC10646330; doi:10.3389/fpubh.2023.1248121)
Supplement: Supplementary file 1 [file Data_Sheet_1.docx]

**Appendix A**


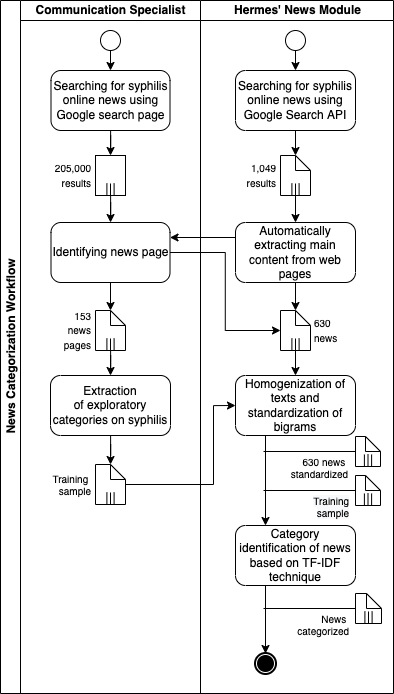


**Figure A1:** News categorization workflow performed in this study.

**Topic A1: Hermes - A Digital Ecosystem to Assess Public Health Policies**

Hermes ecosystem is based on a multidimensional analysis framework which aims to encompass four software products: campaign, communication, education, and epidemiological surveillance.

Campaign works with actions performed within a specific period, on a particular topic (i.e. sexually transmitted infections, alcohol, tobacco, obesity), for a target audience (i.e. men, women, children, homosexuals, sex workers), and how much was spent.

Communication concerns content generated through the Internet, such as: i) online news, ii) interest in a particular topic across search engine query logs, and iii) social network services (SNS).

Education is related to educational courses and research growth. Education is an important indicator to evaluate health professionals, demonstrating scientific interest. The Ministry of Health in Brazil has used Virtual learning platforms that promote Massive Open Online Courses (MOOCs) as lifelong learning strategies for health care workers. The increase in the number of MOOCs on the SUS' Virtual Learning Environment (AVASUS) platform, as well as in the number of trained professionals, are fundamental factors to be assessed. In the area of academics, the number of research and publications can show the relationship between interest in the subject and qualification.

Epidemiological surveillance aims to understand the population awareness regarding behavioral changes. We focus on three directly interconnected variables: i) serological tests, ii) case notification, and iii) medication distribution.

Hermes can obtain the data, detach them according to the specificity, module and granularity of each one, apply machine learning techniques and statistical methods as explained in Panel 2 and show the most relevant information through visual representation. Each stakeholder may then analyze it and draw conclusions relevant to their field or area of interest.

Topic A2: Context and examples to illustrate the seven generic news categories resulting from the thematic analysis.

The **Disease Definition** category was defined based on news items that clearly explain what syphilis is about. Among the selected excerpts, the news mentioned that syphilis is a sexually transmitted infection, curable and disseminated by the bacterium Treponema Pallidum. This information was observed in passages from the news items, such as: “Curable disease, caused by a bacterium and transmitted through sexual intercourse”; “Syphilis is a sexually transmitted infection caused by the bacterium Treponema Pallidum”; “Syphilis is mainly transmitted through unprotected sexual intercourse”; and “It is an infectious disease caused by the bacterium Treponema pallidum. It manifests itself in three stages: primary, secondary and tertiary.”. These news items were broadcast on national news portals, as well as on the websites of the city halls of different municipalities in Brazil.

The **Epidemiological Indicators** category refers to the epidemiological situation of syphilis in the country, mentioning the number of cases of acquired and congenital syphilis based on data provided by the Epidemiological Bulletin of the Ministry of Health. The news selected to illustrate this category featured excerpts such as: “The most recent bulletin from the Ministry of Health, released in October, points out that, in 2018, 26,300 cases of congenital syphilis were registered in the country.”; “Data from the Notifiable Diseases Information System (Sinan) indicate that in Sergipe, the rate of congenital syphilis has decreased in the last four years.”; and “The city of Salvador had a 40% increase in registered cases of syphilis during 2019”. The selected excerpts addressed epidemiological indicators at the national and regional levels, as some of these data were extracted from city hall or state web pages.

The **Prevention** category denotes news that addresses forms of prevention for congenital and acquired syphilis. For example, the excerpts mention the use of condoms and the monitoring of pregnant women during prenatal care. The content that illustrates this category are: “Doctors reinforce the need for condoms during sexual intercourse”; “Syphilis is prevented with the regular use of condoms (female or male) and monitoring of pregnant women.”; “The condom is the flagship and is still the 100% safe device for preventing all STIs”; and “Encouraging the use of condoms among young people as a way to avoid this and other infections.”

The **Rapid Test/Diagnosis** category was defined based on the identification of news that addressed the performance of the rapid test as a form of diagnosis, either just mentioning the test or explaining in more detail how it can be performed, as well as that it is free and made available by the Unified Health System (SUS). In this category, we had: “The test that detects syphilis – and also HIV – is fast, safe and confidential. It is performed by collecting a drop of blood from the fingertip. After 20 minutes, on average, the result comes out.”; “Detection through rapid tests allows for diagnosis, for the patient to take precautions to avoid transmission, and also for early treatment.” and “The Unified Health System provides a quick test to diagnose and treat the disease.”.

The **Consequences** category is related to news that addresses the symptoms of congenital and acquired syphilis. In addition, the news mentions the stages in which syphilis develops and emphasizes that it is a silent disease with few apparent symptoms when it is in its initial stage. Thus, this category can be illustrated as follows: “In adults, the disease has symptoms that can evolve from genital sores and spots on the body, fever, malaise and even lesions on the skin, bones and nervous and cardiovascular systems, and may also develop conditions similar to dementia and depression.”; “Infection in the primary phase has a greater possibility of transmission, and can occur from one person to another during sexual intercourse without a condom, through microscopic wounds or superficial injuries to the mucosa of the vagina or penis.” and “In many cases, the result is the appearance of wounds, discharges, blisters and warts, which can progress to more serious complications such as cancer and even death.”

The **Campaign** category addresses the communication initiatives, advertising campaigns or health promotion identified in the excerpts of the collected news. In this news set, it was found that some of the selected news were publicizing a particular communication campaign to encourage the performance of rapid tests and the prevention of syphilis. To illustrate this category, we have: “The activities are part of the closing of the National Campaign to Combat Syphilis.” The Ministry of Health launched a campaign to encourage the use of condoms among young people as a way to avoid this and other infections.”; and “the Municipal Campaign for the Prevention of Syphilis and Congenital Syphilis, promoted by the Municipal Health Department of Nova Friburgo. The campaign aims to alert the population about the importance of diagnosing and treating Syphilis.”

The **Treatment** category was defined based on news that mentions the term ’treatment’ and clarifies how the sick person can treat the infection. Among the excerpts selected, the following stand out: “Syphilis is treated with antibiotics.”; “The treatment is carried out only with penicillin - the dosage is defined according to the stage.”; and “The treatment is carried out with penicillin, whose Administration scheme varies according to the stage of the disease.”
